# Benzathine penicillin adherence for secondary prophylaxis among patients affected with rheumatic heart disease attending Mulago Hospital

**DOI:** 10.5830/CVJA-2013-022

**Published:** 2013-06

**Authors:** Charles Musoke, Charles Kiiza Mondo, Wanzhu Zhang, Juergen Freers, Emmy Okello, Barbara Kakande, Wilson Nyakoojo

**Affiliations:** Department of Medicine, College of Health Sciences, Makerere University, Kampala, Uganda; Department of Medicine, College of Health Sciences, Makerere University, Kampala, Uganda; Uganda Heart Institute, Mulago National Referral Hospital, Kampala, Uganda; Department of Medicine, College of Health Sciences, Makerere University, Kampala, Uganda; Department of Medicine, College of Health Sciences, Makerere University, Kampala, Uganda; Uganda Heart Institute, Mulago National Referral Hospital, Kampala, Uganda; Uganda Heart Institute, Mulago National Referral Hospital, Kampala, Uganda; Uganda Heart Institute, Mulago National Referral Hospital, Kampala, Uganda

**Keywords:** rheumatic heart disease, benzathine penicillin, secondary prophylaxis, adherence

## Abstract

**Introduction:**

Rheumatic heart disease (RHD) frequently occurs following recurrent episodes of acute rheumatic fever (ARF). Benzathine penicillin (benzapen) is the most effective method for secondary prophylaxis against ARF whose efficacy largely depends on adherence to treatment. Various factors determine adherence to therapy but there are no data regarding current use of benzapen in patients with RHD attending Mulago Hospital. The study aims were (1) to determine the levels of adherence with benzapen prophylaxis among rheumatic heart disease patients in Mulago Hospital, and (2) establish the patient factors associated with adherence and, (3) establish the reasons for missing monthly benzathine penicillin injections.

**Methods:**

This was a longitudinal observational study carried out in Mulago Hospital cardiac clinics over a period of 10 months; 95 consecutive patients who satisfied the inclusion criteria were recruited over a period of four months and followed up for six months. Data on demographic characteristics and disease status were collected by means of a standardised questionnaire and a card to document the injections of benzapen received.

**Results:**

Most participants were female 75 (78.9%). The age range was five to 55 years, with a mean of 28.1 years (SD 12.2) and median of 28 years. The highest education level was primary school for most patients (44, 46.3%) with eight (8.4%) of the patients being illiterate. Most were either NYHA stage II (39, 41.1%) or III (32, 33.7%). Benzathine penicillin adherence: 44 (54%) adhered to the monthly benzapen prophylaxis, with adherence rates ≥ 80%; 38 (46%) patients were classified as non-adherent to the monthly benzapen, with rates less than 80%. The mean adherence level was 70.12% (SD 29.25) and the median level was 83.30%, with a range of 0–100%; 27 (33%) patients had extremely poor adherence levels of ≤ 60%. Factors associated with adherence: higher education status, residing near health facility favoured high adherence, while painful injection was a major reason among poor performers.

**Conclusion:**

The level of non-adherence was significantly high (46%). Residence in a town/city and having at least a secondary level of education was associated with better adherence, while the painful nature of the benzapen injections and lack of transport money to travel to the health centre were the main reasons for non-adherence among RHD patients in Mulago.

## Abstract

In developing countries, rheumatic fever (RF) is the predominant cause of acquired childhood cardiomyopathy.^1^,^2^ The prevalence of RHD is estimated to be higher in developing than in developed countries, ranging from 24/1 000 to 0.3/1 000, respectively.^3^-^5^ Rheumatic heart disease might occur following a single episode of acute rheumatic fever (ARF); however, it is most often the result of recurrent episodes.^6^ Those diagnosed with ARF are at higher risk of suffering further episodes of ARF than the general population, with the incidence of rheumatic fever following streptococcal infection as high as 50% in those with previous ARF,^7^ compared with only 1–3% in the general population.^8^

Long-term treatment with penicillin is recommended to prevent infection with Group A streptococcus among those with a previous diagnosis of ARF, and it has been shown to significantly reduce the morbidity and mortality associated with both recurrent ARF and RHD.^9^,^10^ The severity and prognosis of RHD depends on the extent of cardiac involvement and the frequency of recurrent events. Adherence to penicillin prophylaxis is therefore essential to prevent rapid progression of disease.

Adherence variability to three- or four-weekly injections of benzathine penicillin is well documented, both in the community setting and in hospital-based studies. Several factors could explain the non-adherence observed among these patients: intramuscular injections of benzathine penicillin are painful and may sometimes be associated with allergic reactions.^6^ Among asymptomatic or minimally symptomatic patients, this might prove to be a deterrent, particularly if the links to future recurrence of rheumatic fever are not repeatedly reiterated. Furthermore, practitioners in the community might be reluctant to administer penicillin injections for fear of anaphylaxis.^7^

Other factors which have been identified include: the level of education and training of health workers and/or people with ARF/RHD who may not fully understand the role of secondary prophylaxis in preventing ARF and subsequent heart damage; refusal by some people who do not want to receive treatment despite their level of understanding; difficulties accessing healthcare, that is, travelling to the health facility to receive treatment may be difficult and/or costly, especially for people living in rural and remote areas; forgetting to attend the health centre on the date when secondary prophylaxis is due; staff workloads and priorities. Healthcare staff may be unable to identify and encourage people who do not receive regular secondary prophylaxis.^11^,^12^

In Uganda, RHD is the second leading cause of acquired heart disease after hypertensive heart disease.^13^ Current data regarding adherence rates to secondary benzathine penicillin prophylaxis among these patients are unknown despite our knowledge that good adherence is protective for severe forms of RHD. Therefore, the study aims were (1) to determine the level of adherence to benzathine penicillin prophylaxis among rheumatic heart disease patients attending Mulago Hospital, (2) establish the patient factors associated with adherence and, (3) establish the reasons for missing monthly benzathine penicillin injections.

## Methods

Institutional ethics approval was obtained from the School of Medicine Research and the Ethics Committee of the College of Health Sciences, Makerere University. We obtained informed consent for all the patients and informed assent for those unable to give consent. Patients’ initials and study numbers were put on the questionnaires instead of full names to ensure confidentiality.

This was a longitudinal observational study carried out in Mulago Hospital, the national referral hospital, and Makerere University teaching hospital located in Kampala, Uganda, which receives more than 250 patients with RHD annually. The target population included all patients clinically diagnosed with rheumatic heart disease and confirmed by echocardiography, as previously described.^5^

New and old RHD patients aged five to 55 years who were eligible to continue prophylaxis for a period not less than one year from the time of recruitment and consented to the study were recruited. Each patient was then given a benzathine penicillin prophylaxis card recommending the appropriate monthly (four-weekly) dose of benzathine penicillin according to the Uganda clinical guidelines, which recommends 2.4 MU for adults, 0.6 MU for children ≤ 30 kg and 1.2 MU for those > 30 kg.^8^ Patients with known allergy to benzathine penicillin were excluded from the study.

**Fig. 1.**
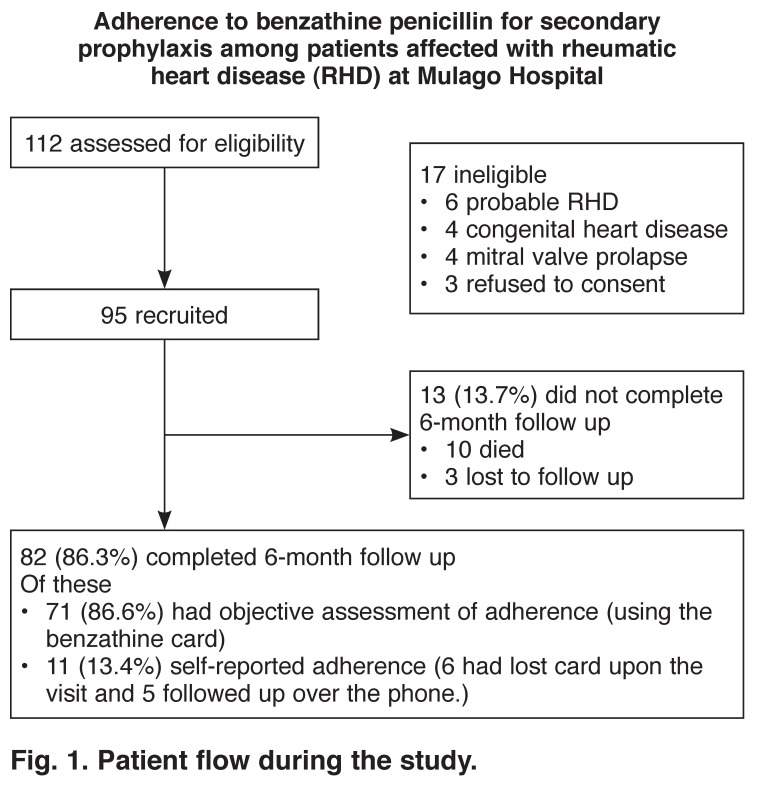
Patient flow during the study.

Patients who met the inclusion criteria were consecutively recruited over a period of four months until a total of 95 patients was reached (Fig. 1). An identification number or unique patient number (UPN) was assigned to each consenting patient. For those who refused to consent, the reason for refusal was documented in the study book.

A focused clinical assessment was done using a standardised pre-tested questionnaire in which the socio-demographic data, details of physical findings, and details of findings on the electrocardiogram and at echocardiography were recorded. In addition, data regarding the following were collected: personal history of hypertension, diabetes, stroke and other heart diseases. Socio-economic factors recorded were educational level, occupation, and total income (of parents in the case of children or students).

For each patient recruited, information regarding the importance of secondary prophylaxis was provided as part of the whole information package given to the RHD registry patients, including all their other treatment modalities. This was done in liaison with the primary attending clinicians. This was to help capture the dates and signatures of the health workers where the patient received the benzathine penicillin injections over the following six months. The card had the name of the patient, which would help track the patients’ UPN through the study book.

For purposes of limiting loss to follow up, data concerning the following were collected: the patients’ phone numbers if available/number of the caretaker for children; phone numbers of at least two close relatives or friends, which would be tested at the time of recording to ascertain their existence; the number for the principle investigator was written at the back of each patient’s benzathine penicillin card, and patients were urged to call and inform the principle investigator if they were planning to change their phone numbers.

After recruitment, each patient was told to continue attending his/her regular clinic, as scheduled by the primary care clinician, and he/she was to be reviewed in the general RHD registry every three months by other registry clinicians. For this particular study, the patients were reviewed again at the end of six months’ follow up.

At the six-month follow-up visit, patients were contacted by phone and were encouraged to come with their benzathine penicillin prophylaxis cards so that data regarding their rates of adherence could be extracted. Those who were unable to travel at the six-month follow for various reasons were requested to read off the number of injections received at the time of the call from the card, and this was recorded in their follow-up questionnaire. These patients were nevertheless encouraged to take time off and come to the clinic on any other appropriate time for follow-up care.

For those patients who had lost the benzathine prophylaxis card, we relied on self-reports as to how many injections he/she had received over the previous six months. The proportion of these patients, together with those from whom information was obtained over the phone was catered for during analysis. At six months of follow up, a separate structured, pre-coded questionnaire was administered to those patients without 100% adherence, with the aim of capturing the factors or reasons for missing the benzathine prophylaxis injections.

Measuring benzathine penicillin injection delivery^9^ was calculated as a percentage of the number of injections received, divided by the number prescribed and multiplied by 100. Receiving less than 80% of injections places an individual at a higher risk of recurrent ARF.^9^

## Statistical analysis

Data were entered in Epidata 3.1, backed and cleaned to prevent data loss and then exported to STATA version 10.0. Continuous variables were summarised in means (standard deviation) and median (interquartile range). Categorical data were summarised using frequency and percentages and results are presented in tables. To address the first objective, the adherence rates for RHD patients attending Mulago Hospital were calculated as follows:

The number of injections required in six months for a patient on four-weekly benzathine prophylaxis = six injections. Adherence rates for individual patients was calculated as:

$\frac{no\;of\;injections\;received}{no\;of\;injections\;expected}\;\times 100\%$

We then proceeded to determine the level of adherence, the mean adherence rate and median.

For the second objective, bivariate analysis was done with a confidence interval of 95%, and Pearson’s chi-square test was used to ascertain statistical significance. Variables included in the bivariate analysis included age, gender, patient’s home address, level of education, patient’s employment status, the NYHA class, tribe, and history of previous use of benzathine penicillin (all variables were put in two categories). Fisher’s exact test was used where cells had less than five readings. A *p*-value < 0.05 was considered significant. To ascertain for statistical significance (*p* < 0.05) between subjective and objective assessment for adherence, the Pearson’s chi-square test was used.

## Results

From June 2011 to March 2012, out of the 112 patients screened for eligibility, 95 rheumatic heart disease patients were recruited and followed up for a period of six months to assess their adherence levels and associated factors (Fig. 1). Reasons for excluding the 17 patients included: six patients with probable RHD, four patients with congenital heart disease, four with mitral valve prolapse and three declined to consent. Out of these 95 patients, 82 (86.3%) completed the six-month follow-up period; 13 (13.7%) did not complete six months of follow up because 10 had died and three were lost to follow up. Of the 82 patients who completed follow up, 71 (86.6%) were objectively assessed for adherence levels using the benzathine penicillin card provided at the beginning of the study and 11 (13.4%) were subjectively assessed using self-reporting. Of the 11 patients, six came to hospital at follow up but had lost their cards and five were followed up over the phone.

(Table 1)Table 1 shows the baseline characteristics of the patients. The majority was female (75, 78.9%). The patients’ ages ranged from five to 55 years, with a mean age of 28.1 years (SD 12.2) and median 28 years. The majority of patients were over 18 years (73, 76.8%) and 57 (60%) patients were town/city residents compared to 38 (40%) from rural areas. The majority (44, 46.3%) had primary educational level while eight (8.4%) were illiterate. Most of the patients were either NYHA class II (39, 41.1%) or III (32, 33.7%). The majority (49, 51.6%) were in the Baganda tribe.

**Table 1:** Baseline Characteristics Of The Patient

*Variable*	*Frequency (n = 95)*	*Percentage (%)*
Age		
< 18 years	22	23.2
≥18 years	73	76.8
Patients’ home		
Rural	38	40.0
Town/city	57	60.0
Gender		
Female	75	78.9
Male	20	21.1
Level of education		
College	10	10.5
Secondary	29	30.5
Vocational	4	4.2
Primary	44	46.3
None	8	8.4
Employment status		
Unemployed	65	68.4
Employed	30	31.6
NYHA class		
I	7	7.4
II	39	41.1
III	32	33.7
IV	17	17.9
Tribe		
Ganda	49	51.6
Soga	4	4.2
Toro	2	2.1
Ankole	9	9.5
Nyoro	2	2.1
Acholi	3	3.2
Others	26	27.4

**Fig. 2.**
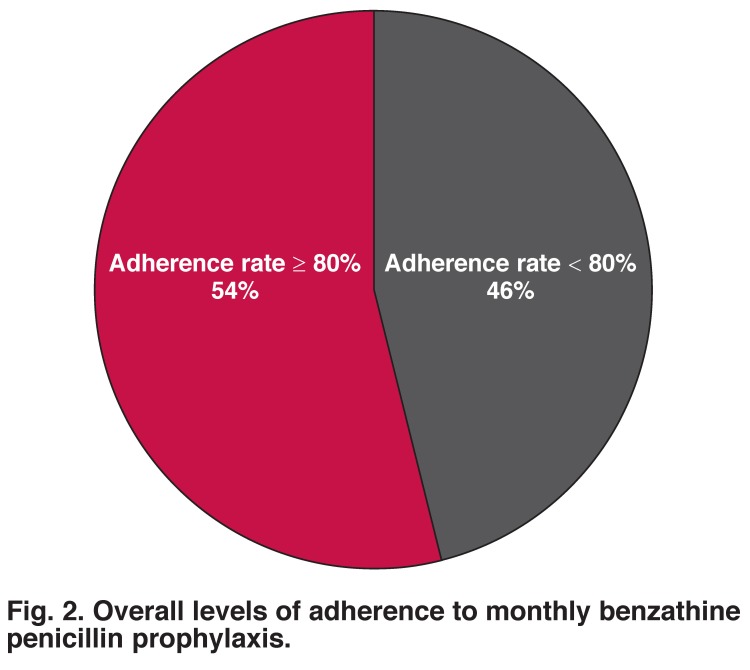
Overall levels of adherence to monthly benzathine penicillin prophylaxis.

**Fig. 3.**
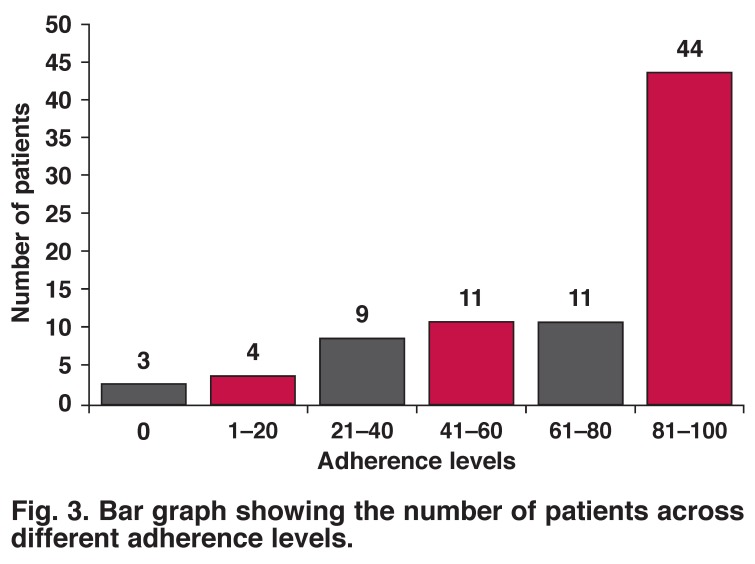
Bar graph showing the number of patients across different adherence levels.

Fig. 2 shows the levels of adherence after six months of follow up. Of the 82 patients who completed the six-month follow up, 44 (54%) had adhered to the monthly benzathine penicillin prophylaxis, with adherence rates ≥ 80%; 38 (46%) patients were classified as non-adherent to the monthly benzathine penicillin, with rates less than 80%. The mean adherence rate was 70.12% (SD 29.25) and the median rate was 83.30% with a range of 0–100%. Fig. 3 shows the number of patients across different adherence levels; 27 (33%) of the patients had extremely poor adherence rates of ≤ 60%.

Of the 82 patients who completed the six-month follow up, 71 (86.6%) presented their cards at follow up and their adherence rates were assessed objectively by counting the number of injections received over the follow-up time, compared to the 11(13.4%) who gave a self-report of the number of injections received. Table 2 shows a lack of significant difference between adherence levels as measured among these two groups of patients.

**Table 2:** Comparison Between Objective And Subjective Assessment Of Adherence

*Variable*	*< 80% adherence (n =38)*	*≥ 80% adherence (n =44)*	*OR (95% CI)*	p*-value*
Came with card	32 (84.2)	39 (88.6)	0.684 (0.191–2.448)	0.558
Self report	6 (15.8)	5 (11.4)		

Table 3 shows the patient factors associated with adherence to the monthly benzathine penicillin prophylaxis. Although statistically significant associations with adherence were not found, trends towards adherence were demonstrated among patients who resided in a town/city (OR 1.73; CI 0.64–4.72) and those with at least secondary levels of education (OR 2.21; CI 0.83–5.93). There was no difference in the levels of adherence between those under 18 compared with ≥18 years. The lack of previous exposure to benzathine penicillin was not associated with better adherence.

**Table 3:** Patient Factors Associated With Adherence To Monthly Benzathine Penicillin Prophylaxis

*Variable*	*≥ 80% adherence n = 44 (%)*	*< 80% adherence n = 38 (%)*	*OR (95% CI)*	p*-value*
Age				
< 18 years	10 (22.7)	8 (21.1)	1.10 (0.34–3.58)	0.855
≥ 18 years	34 (77.3)	30 (78.9)		
Patients’ home				
Town/city	30 (68.2)	21(55.3)	1.73 (0.64–4.72)	0.229
Rural	14 (31.8)	17 (44.7)		
Gender				
Female	33(75.0)	33(86.8)	0.45 (0.12–1.63)	0.177
Male	11(25.0)	5(13.2)		
Level of education^1^				
Secondary and above	26(59.1)	15 (39.5)	2.21 (0.83–5.93)	0.076
Less than secondary	18 (40.9)	23 (60.5)		
Employment status^2^				
Currently employed	13(29.5)	13(34.2)	0.81 (0.29–2.26)	0.651
Unemployed	31(70.5)	25(65.8)		
NYHA stage				
I and II	22(50.0)	21(55.3)	0.81 (0.31–2.12)	0.634
III and IV	22(50.0)	17(44.7)		
Tribe				
Ganda	21(47.7)	19(50.0)	0.91 (0.35–2.38)	0.837
Other	23(52.3)	19 (50.0)		
Previous use of benzathine^3^				
No	41(93.2)	37(97.4)	0.37 (0.01–4.28)	0.380
Yes	3(6.8)	1(2.6)		

1Employment status for the attendants was considered for children and students. 2For patients < 13 years, education level of primary caretaker was considered. 3Fisher’s exact test was used. OR = odds ratio, CI = confidence interval.

Table 4 shows the commonest reasons by the respondents for missing a benzathine penicillin dose. These reasons included those given by patients who had adherence levels above 80% but not reaching 100%. The commonest reason for missing a dose was the painful nature of the benzathine penicillin injection, reported by 27 respondents (29% of all reasons given). This was closely followed by lack of transport money to the health facility to receive the injection. The other reasons included injection abscesses, attendant too busy at home with children and unable to go for the injection, they thought it was acceptable to miss a few times, one patient had valvular surgical repair and was advised by the local health practitioner that there was no need for any more injections.

**Table 4:** Reasons For Missing Monthly Benzathine Prophylaxis Injections

*Reasons for missing*	*Frequency*	*Percentage of responses (93 responses)*
Injection painful	27	29
Did not have transport money	25	26.9
Felt healthy and well	11	11.8
Away from home	7	7.5
Friends advised me otherwise	4	4.3
Felt sick and unable to take the injection	3	3.2
Other	16	17.2
Total	93	100

## Discussion

A patient with rheumatic heart disease is expected to receive at least 80% of the annual prescribed injections. Receiving less than 80% of the injections places an individual at a higher risk of recurrent ARF and its complications.^9^ In this study, adherence was considered as when a patient had received at least 80% of the required injections over a period of six months.

An adherence level of at least 80% was found among 44 (54%) patients, compared to 38 (46%) with adherence levels less than 80%. The mean adherence was determined at 70.12% (SD 29.25). This was similar to the adherence level determined by Harrington in an aboriginal community in Australia, in which 59% of patients had received more than 75% of their prescribed injections during an interview.^14^

However, the level of adherence we determined in this study was considerably higher than that found among RHD patients in another Aboriginal community in Australia were the mean adherence level was 56% when patients were followed up for a period of 24 months.^15^ On the other hand, this level of adherence was considerably less than that found in several other studies such as the study done in Haryana district in India, which found that 90% of the patients had received over 80% of their benzathine injections over the previous eight years.^16^

The variability in levels of adherence may reflect the different systems in which these studies were done, duration of follow up, the different factors that may influence adherence, the individual study designs, and the different cut-off points for defining adherence in the different studies. This variability is still hard to explain confidently since low levels of adherence have been demonstrated in Australia were rheumatic heart disease registries exist and are fully functional. However, given all these factors, the level of adherence as was determined in this study was low, because it placed a significant proportion of patients (46%) at risk of recurrent episodes of ARF and worsening of valvular heart lesions, with resultant poor prognosis.

Analysing the association of patient factors with adherence levels provides insight into those groups at particular risk of recurrence through poor adherence. No particular patient factor was found to be significantly associated with adherence. This was not surprising as a similar study by Stewart in Australia found no significant demographic factors associated with adherence.^15^ However, trends towards adherence were demonstrated among patients who resided in a town/city and those with at least secondary level of education.

The fact that patients who resided in a town/city tended to have better adherence could be explained by the fact that these patients have easier access to healthcare facilities compared to those from rural areas. This finding could be supported by a study done by Kathie Walker, who found that patients who stayed far from the health facilities (> 10 km) were significantly associated with poor compliance.^11^ There was no difference between men and women regarding their level of adherence, although an earlier study by Dorothy^12^ had revealed that men are more likely to be non-adherent compared to their female counterparts.

Whether the lack of significant factors reflects a true lack of association, a limited time to follow up, or rather, the effect of a small sample size is uncertain from these results. Most of the studies done on this topic have not analysed for these patient factors, making it rather a complex area to discuss. Nevertheless, these trends do identify subgroups that might be at increased risk of recurrent ARF and worsening of RHD through non-adherence.

The commonest reason reported for missing monthly benzathine prophylaxis injections was the painful nature of the injection (27, 29%). This was closely followed by lack of money (26.9%) and the fact that the patients felt healthy and well (11.8%). These factors have also been described by WHO expert consultation in Geneva.^6^ Despite some reports which have indicated that forgetting could be an import reason for missing injections, it did not feature in this study. This could have been due to the benzathine penicillin card but it cannot be ascertained for sure, since no control group existed. However, other factors of interest included development of injection abscesses, misperceptions by the local health worker that a patient does not need prophylaxis after heart valve surgery, and missing an injection during admission. These factors will form the basis for intervention in order to improve adherence among our patients.

## Limitations

Providing a card, which is not routinely done in the normal setting, may have improved adherence rates as this could have acted as a reminder to go for the injection although the card was devoid of reminding dates. In our setting where records are poor, we could not find any other objective way to measure these rates. The results obtained may not apply to the general population because of the sampling procedure used. The follow-up time of six months may have been too short to accurately assess the levels of adherence since adherence has been shown to decline with time in some studies.

## Conclusions

Although the mean level of adherence was fairly good, the level of non-adherence among these rheumatic heart disease patients was significant. Although no particular patient factor was found to be significantly associated with adherence, we determined that residing in a town/city and having at least a secondary school level of education was associated with a trend towards adherence. The painful nature of the benzathine penicillin injections and lack of transport money to travel to the health centre were the main reasons for non-adherence among RHD patients attending Mulago Hospital.
